# Supplementary material for: Costly mistakes: Why and when spelling errors in resumes jeopardise interview chances
Source: PLoS One. 2023 Apr 5;18(4):e0283280. doi: 10.1371/journal.pone.0283280 (PMC10075394; doi:10.1371/journal.pone.0283280)
Supplement: S1 File — (DOCX) [file pone.0283280.s001.docx]

# Appendix

[Appendix Figure 1]

[Appendix Figure 2]

[Appendix Table 1]

[Appendix Table 2]

[Appendix Table 3]

[Appendix Table 4]

[Appendix Table 5]

[Appendix Table 6]

| **Language error (in Dutch)** |
| --- |
| Diplomma |
| Sekundair |
| Behaalt |
| Geslaagt |
| Programa |
| Adminiestratief |
| Magasijnier |
| Horeka |
| Bakerij |
| Vollybal |
| Hocky |
| Vrijwilligerwerk |
| Afgestudeert |
| Belgishe |
| Manlijk/Vrouwlijk |
| Extraas |
| Excelente |
| Telefoonummer |
| Edukatie |
| Uitstekkende |
| PC-programas |
| Beschikkent |
| Curicculair |
| PC-/Taalkenis |

Appendix Figure 1. Overview of potential spelling errors used in the Dutch experimental materials.


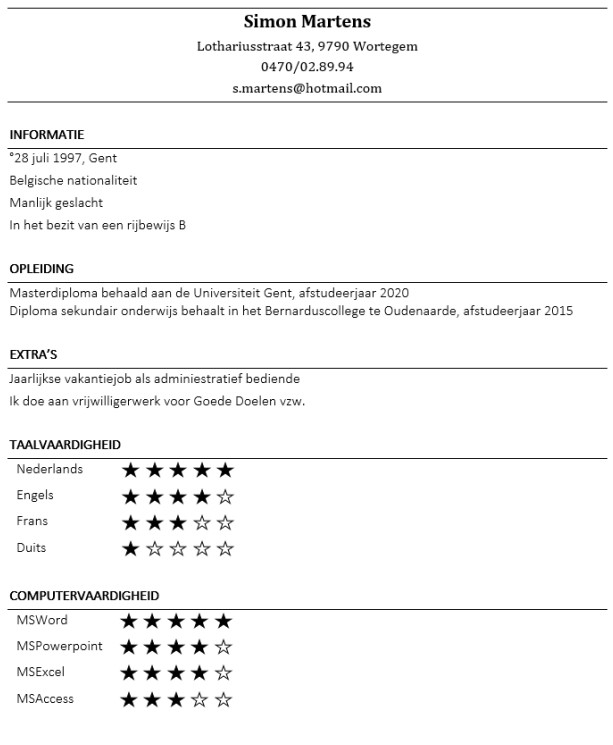


Appendix Figure 2. Example Dutch vignette used in the experiment.

| Appendix Table 1. Jobs and corresponding job characteristics used in the experiment | | | |
| --- | --- | --- | --- |
| **Job** | **Req. level of education** | **Sector** | **Req. written communication skills** |
| Recreation worker | Low | Services | Low |
| Production worker | Low | Manufacturing | Low |
| Secretary | Low | Services | High |
| Assistant graphic designer | Low | Manufacturing | High |
| Specialist electronics | High | Manufacturing | Low |
| Air traffic controller | High | Services | Low |
| Human resources manager | High | Services | High |
| Audiovisual specialist | High | Manufacturing | High |
| Notes. Abbreviation used: req. (required). Jobs were selected and categorised based on data provided by O*Net, as described in Subsection 2.3. | | | |

| Appendix Table 2. Regression results with interview probability as the outcome variable, two-way interactions included for two spelling errors | | | | | |
| --- | --- | --- | --- | --- | --- |
|  | **Interview probability** | | | | |
|  | **(1)** | **(2)** | **(3)** | **(4)** | **(5)** |
| A. APPLICANT CHARACTERISTICS | | | | | |
| Spelling errors (ref. = none) |  |  |  |  |  |
| Two errors | −0.730*** (0.135) | 0.008 (0.536) | −0.801*** (0.206) | −0.836 (0.813) | −0.325 (0.963) |
| Five errors | −1.850*** (0.155) | −1.878*** (0.158) | −1.847*** (0.155) | −1.849*** (0.155) | −1.871*** (0.158) |
| Female | 0.375*** (0.121) | 0.232 (0.157) | 0.375*** (0.120) | 0.374*** (0.121) | 0.233 (0.157) |
| Age of graduation (ref. = foreseen age) |  |  |  |  |  |
| One year later | 0.164 (0.146) | 0.333 (0.213) | 0.148 (0.146) | 0.162 (0.147) | 0.321 (0.214) |
| Two years later | 0.137 (0.143) | 0.317 (0.215) | 0.135 (0.142) | 0.138 (0.143) | 0.313 (0.215) |
| Student work (ref. = none mentioned) |  |  |  |  |  |
| On the weekends | 0.308** (0.145) | 0.244 (0.216) | 0.316** (0.146) | 0.308** (0.145) | 0.239 (0.217) |
| During holidays | 0.227* (0.132) | 0.419* (0.204) | 0.238* (0.133) | 0.229* (0.133) | 0.427** (0.205) |
| Hobbies (ref. = none mentioned) |  | | | | |
| Team sports | 0.308** (0.141) | 0.400* (0.219) | 0.305** (0.141) | 0.309** (0.142) | 0.397* (0.220) |
| Volunteering | 0.706*** (0.139) | 0.935*** (0.209) | 0.709*** (0.139) | 0.710*** (0.140) | 0.937*** (0.210) |
| Achievement in tert. edu. (ref. = none mentioned) |  |  |  |  |  |
| Graduated cum laude | 0.124 (0.214) | 0.112 (0.262) | 0.119 (0.211) | 0.114 (0.217) | 0.047 (0.294) |
| International experience | 0.438** (0.213) | 0.589** (0.270) | 0.445** (0.211) | 0.432** (0.213) | 0.527* (0.297) |
| Mother tongue perceived as excellent | 0.155 (0.128) | 0.213 (0.152) | 0.153 (0.129) | 0.155 (0.129) | 0.217 (0.152) |
| Two errors × Female |  | 0.456 (0.322) |  |  | 0.443 (0.324) |
| Two errors × Graduated one year later |  | −0.593 (0.430) |  |  | −0.596 (0.431) |
| Two errors × Graduated two years later |  | −0.686 (0.425) |  |  | −0.674 (0.440) |
| Two errors × Student work on the weekends |  | 0.011 (0.424) |  |  | 0.034 (0.426) |
| Two errors × Student work during holidays |  | −0.666 (0.439) |  |  | −0.653 (0.440) |
| Two errors × Team sports |  | −0.204 (0.450) |  |  | −0.213 (0.454) |
| Two errors × Volunteering |  | −0.454 (0.415) |  |  | −0.456 (0.416) |
| Two errors × Graduated cum laude (tert. edu.) |  | −0.061 (0.427) |  |  | 0.096 (0.548) |
| Two errors × International experience (tert. edu.) |  | −0.461 (0.458) |  |  | −0.286 (0.585) |
| Two errors × Mother tongue perceived as excellent |  | −0.093 (0.298) |  |  | −0.107 (0.303) |
|  | | | | | |
| Req. level of education: high | 0.040 (0.274) | 0.050 (0.274) | 0.116 (0.292) | 0.044 (0.275) | 0.110 (0.311) |
| Req. written communication: high | −0.649*** (0.222) | −0.656*** (0.221) | −0.662*** (0.235) | −0.649*** (0.223) | −0.670*** (0.236) |
| Req. type of labour: service | 0.023 (0.216) | 0.008 (0.216) | −0.088 (0.232) | 0.021 (0.216) | −0.095 (0.235) |
| Two errors × high level of education req. |  |  | −0.228 (0.263) |  | −0.171 (0.401) |
| Two errors × high written communication req. |  |  | 0.046 (0.247) |  | 0.050 (0.260) |
| Two errors × service labour req. |  |  | 0.309 (0.242) |  | 0.285 (0.249) |
| C. PARTICIPANT CHARACTERISTICS |  | | | | |
| Female | 0.139 (0.223) | 0.129 (0.224) | 0.137 (0.223) | 0.059 (0.239) | 0.057 (0.239) |
| Age (c) | −0.001 (0.009) | 0.001 (0.009) | −0.001 (0.009) | −0.001 (0.011) | −0.005 (0.011) |
| Language sensitivity (c) | −0.110 (0.068) | −0.113 (0.069) | −0.109 (0.068) | −0.108 (0.073) | −0.111 (0.074) |
| Two errors × Female |  |  |  | 0.225 (0.252) | 0.192 (0.249) |
| Two errors × Age |  |  |  | 0.001 (0.011) | 0.003 (0.011) |
| Two errors × Language sensitivity |  |  |  | −0.006 (0.077) | −0.004 (0.077) |
| N | 1,335 | | | | |
| Notes. Abbreviations used: c (continuous variable), ref. (reference category), req. (required), and tert. edu. (tertiary education). The presented statistics are coefficient estimates and their standard errors in parentheses. Standard errors are corrected for clustering of the observations at the participant level. ***, ** and * indicate significance at the 1%, 5%, and 10% levels, respectively. | | | | | |

| Appendix Table 3: Mediation analysis with interview probability as the outcome and three mediators | | | | |
| --- | --- | --- | --- | --- |
|  | **Mediators** |  |  | **Interview probability** |
|  | **Perceived**  **interpersonal skills** | **Perceived conscientiousness** | **Perceived**  **mental abilities** |  |
| A. APPLICANT CHARACTERISTICS |  |  |  |  |
| Spelling errors (ref. = none) |  |  |  |  |
| Two errors | −0.228*** (0.084) | −0.427*** (0.085) | −0.399*** (0.076) | −0.342*** (0.108) |
| Five errors | −0.745*** (0.096) | −1.027*** (0.099) | −1.000*** (0.096) | −0.825*** (0.119) |
| Female | 0.425*** (0.075) | 0.402*** (0.072) | 0.330*** (0.070) | −0.025 (0.099) |
| Age of graduation (ref. = foreseen age) |  |  |  |  |
| One year later | 0.089 (0.091) | 0.254*** (0.095) | 0.161* (0.092) | −0.008 (0.106) |
| Two years later | 0.021 (0.082) | 0.123 (0.087) | 0.051 (0.084) | 0.074 (0.102) |
| Student work (ref. = none mentioned) |  |  |  |  |
| On the weekends | 0.331*** (0.084) | 0.468*** (0.085) | 0.204*** (0.078) | −0.003 (0.114) |
| During holidays | 0.172** (0.084) | 0.363*** (0.085) | 0.142* (0.081) | 0.019 (0.095) |
| Hobbies (ref. = none mentioned) |  |  |  |  |
| Team sports | 0.494*** (0.079) | 0.230*** (0.086) | 0.275*** (0.078) | −0.045 (0.107) |
| Volunteering | 0.711*** (0.081) | 0.548*** (0.087) | 0.484*** (0.080) | 0.103 (0.108) |
| Achievement in tert. edu. (ref. = none mentioned) |  |  |  |  |
| Graduated cum laude | 0.139 (0.134) | 0.186 (0.138) | 0.392*** (0.130) | −0.186 (0.171) |
| International experience | 0.395*** (0.123) | 0.334*** (0.130) | 0.590*** (0.126) | −0.092 (0.167) |
| Mother tongue perceived as excellent | 0.055 (0.076) | 0.043 (0.078) | 0.131* (0.074) | 0.052 (0.095) |
| B. JOB REQUIREMENTS |  |  |  |  |
| Req. level of education: high | −0.191 (0.160) | −0.189 (0.166) | 0.115 (0.163) | 0.066 (0.218) |
| Req. written communication: high | −0.094 (0.128) | −1.132 (0.131) | −0.186 (0.129) | −0.485*** (0.183) |
| Req. type of labour: service | 0.096 (0.126) | −0.041 (0.128) | −0.181 (0.123) | 0.110 (0.180) |
| C. PARTICIPANT CHARACTERISTICS |  |  |  |  |
| Female | 0.170 (0.125) | 0.018 (0.132) | 0.090 (0.127) | 0.033 (0.181) |
| Age (c) | −0.009 (0.006) | −0.008 (0.006) | −0.005 (0.006) | 0.007 (0.008) |
| Language sensitivity (c) | 0.015 (0.036) | −0.015 (0.035) | −0.057 (0.037) | −0.078 (0.056) |
| D. MEDIATING PERCEPTIONS |  |  |  |  |
| Perceived interpersonal skills (s) |  |  |  | 0.289*** (0.089) |
| Perceived conscientiousness (s) |  |  |  | 0.205** (0.097) |
| Perceived mental abilities (s) |  |  |  | 0.590*** (0.099) |
| N | 1,335 |  |  |  |
| Notes. Abbreviations used: s (scale consisting of multiple items), ref. (reference category), req. (required), and tert. edu. (tertiary education). The presented statistics are coefficient estimates and their standard errors in parentheses for the mediation model outlined in Subsection 3.2. Standard errors are corrected for clustering of the observations at the participant level. ***, ** and * indicate significance at the 1%, 5%, and 10% levels, respectively. | | | | |

| Appendix Table 4. Robustness checks of mediation analysis: percentages of spelling errors’ effects on hiring outcomes explained by mediators | | | | | | |
| --- | --- | --- | --- | --- | --- | --- |
| **Mediators** | **Hiring probability as alternative outcome** | | **Subsample with low or average social desirability** | | **Subsample with hiring tenure of at least one year** | |
|  | **Two spelling errors** | **Five spelling errors** | **Two spelling errors** | **Five spelling errors** | **Two spelling errors** | **Five spelling errors** |
|  | **Percentage of spelling error’s effect on hiring probability explained by mediators [p-value]** | | **Percentage of spelling error’s effect on interview probability explained by mediators [p-value]** | | **Percentage of spelling error’s effect on interview probability explained by mediators [p-value]** | |
| Perceived interpersonal skills (s) | **9.0** [0.038] | **11.6** [0.033] | 11.2 [0.080] | **12.9** [0.007] | 8.8 [0.062] | **11.6** [0.004] |
| Perceived conscientiousness (s) | **12.1** [0.049] | **11.9** [0.001] | 12.9 [0.070] | 11.6 [0.058] | **12.5** [0.048] | **12.5** [0.048] |
| Perceived mental abilities (s) | **32.2** [0.000] | **31.9** [0.001] | **29.6** [0.001] | **28.0** [0.001] | **30.8** [0.001] | **30.8** [0.001] |
| N | 1,335 | | 1,035 | | 1,260 | |
| Notes. Abbreviation used: s (scale consisting of multiple items). P-values are corrected for clustering of observations at the participant level. Percentages related to p-values below 5% are in bold. Observations are categorised as ‘low or average social desirability’ if recruiters scored socially desirable answering tendencies below the sample mean increased by one standard deviation. | | | | | | |

| Appendix Table 5: Extended mediation analysis with interview probability as the outcome and perception items as mediators | | | | | |
| --- | --- | --- | --- | --- | --- |
|  | **Mediators** |  |  |  | **Interview probability** |
|  | **Perceived quality of communication** | **Perceived quality of communication during a job interview** | **Perceived ability to get along with others in the job.** | **Perceived pleasure in interaction** |  |
| A. APPLICANT CHARACTERISTICS |  |  |  |  |  |
| Spelling errors (ref. = none) |  |  |  |  |  |
| Two errors | −0.334*** (0.100) | −0.364*** (0.102) | −0.044 (0.096) | −0.169* (0.089) |  |
| Five errors | −0.970*** (0.121) | −1.037*** (0.112) | −0.380*** (0.104) | −0.592*** (0.100) |  |
| Female | 0.544*** (0.093) | 0.511*** (0.085) | 0.314*** (0.086) | 0.331*** (0.079) |  |
| Age of graduation (ref. = foreseen age) |  |  |  |  |  |
| One year later | 0.043 (0.114) | 0.719 (0.109) | 0.067 (0.098) | 0.175* (0.095) |  |
| Two years later | 0.001 (0.104) | 0.063 (0.099) | −0.106 (0.091) | 0.126 (0.088) |  |
| Student work (ref. = none mentioned) |  |  |  |  |  |
| On the weekends | 0.456*** (0.106) | 0.350*** (0.099) | 0.294*** (0.097) | 0.223** (0.089) |  |
| During holidays | 0.290*** (0.107) | 0.190* (0.099) | 0.112 (0.096) | 0.095 (0.087) |  |
| Hobbies (ref. = none mentioned) |  |  |  |  |  |
| Team sports | 0.448*** (0.101) | 0.472*** (0.096) | 0.600*** (0.091) | 0.456*** (0.087) |  |
| Volunteering | 0.736*** (0.098) | 0.597*** (0.097) | 0.897*** (0.095) | 0.614*** (0.089 |  |
| Achievement in tert. edu. (ref. = none mentioned) |  |  |  |  |  |
| Graduated cum laude | 0.175 (0.157) | 0.190 (0.155) | 0.125 (0.148) | 0.066 (0.143) |  |
| International experience | 0.432*** (0.150) | 0.347** (0.149) | 0.327** (0.136) | 0.474*** (0.140) |  |
| Mother tongue perceived as excellent | 0.198 (0.95) | 0.109 (0.091) | 0.023 (0.081) | 0.069 (0.079) |  |
| B. JOB REQUIREMENTS |  |  |  |  |  |
| Req. level of education: high | −0.284 (0.176) | −0.084 (0.180) | −0.161 (0.170) | −0.235 (0.170) |  |
| Req. written communication: high | −0.066 (0.139) | −0.115 (0.143) | −0.181 (0.134) | −0.015 (0.137) |  |
| Req. type of labour: service | −0.043 (0.139) | 0.126 (0.140) | 0.183 (0.131) | 0.118 (0.133) |  |
| C. PARTICIPANT CHARACTERISTICS |  |  |  |  |  |
| Female | 0.216 (0.138) | 0.210 (0.142) | 0.088 (0.129) | 0.167 (0.133) |  |
| Age (c) | −0.005 (0.007) | −0.006 (0.007) | −0.011* (0.006) | −0.015** (0.006) |  |
| Language sensitivity (c) | −0.004 (0.038) | −0.033 (0.041) | 0.060 (0.041) | 0.038 (0.041) |  |
| D. MEDIATING PERCEPTIONS |  |  |  |  |  |
| Perceived quality of communication |  |  |  |  | 0.093 (0.079) |
| Perceived quality of communication during a job interview |  |  |  |  | 0.228*** (0.077) |
| Perceived ability to get along with others in the job |  |  |  |  | −0.104* (0.061) |
| Perceived pleasure in interaction |  |  |  |  | 0.129* (0.071) |
| N | 1,335 |  |  |  |  |
| Notes. Abbreviations used: s (scale consisting of multiple items), ref. (reference category), req. (required), and tert. edu. (tertiary education). The presented statistics are coefficient estimates and their standard errors in parentheses for the mediation model outlined in Subsection 3.2. Standard errors are corrected for clustering of the observations at the participant level. ***, ** and * indicate significance at the 1%, 5%, and 10% levels, respectively. | | | | | |

| Appendix Table 5 continued (1): Extended mediation analysis with interview probability as the outcome and perception items as mediators | | | | | | |  |
| --- | --- | --- | --- | --- | --- | --- | --- |
|  | **Mediators** |  |  |  |  | **Interview probability** |  |
|  | **Perceived as hard-working** | **Perceived as organised** | **Perceived as thorough** | **Perceived as being responsible** | **Perceived as systematic** |  |  |
| 1. APPLICANT CHARACTERISTICS |  |  |  |  |  |  |  |
| Spelling errors (ref. = none) |  |  |  |  |  |  |  |
| Two errors | −0.226** (0.105) | −0.417*** (0.100) | −0.770*** (0.107) | −0.312*** (0.095) | −0.413*** (0.096) |  |  |
| Five errors | −0.589*** (0.107) | −1.066*** (0.117) | −1.622*** (0.125) | −0.932*** (0.116) | −1.150*** (0.113) |  |  |
| Female | 0.308*** (0.082) | 0.429*** (0.086) | 0.575*** (0.095) | 0.346*** (0.084) | 0.352*** (0.084) |  |  |
| Age of graduation (ref. = foreseen age) |  |  |  |  |  |  |  |
| One year later | 0.242** (0.108) | 0.278** (0.111) | 0.246** (0.122) | 0.272** (0.106) | 0.231** (0.111) |  |  |
| Two years later | 0.116 (0.096) | 0.172* (0.103) | 0.172 (0.116) | 0.617 (0.096) | 0.140 (0.103) |  |  |
| Student work (ref. = none mentioned) |  |  |  |  |  |  |  |
| On the weekends | 0.949*** (0.103) | 0.401*** (0.101) | 0.278** (0.109) | 0.465** (0.098) | 0.246** (0.100) |  |  |
| During holidays | 0.614*** (0.096) | 0.376*** (0.102) | 0.195* (0.111) | 0.388*** (0.097) | 0.242** (0.102) |  |  |
| Hobbies (ref. = none mentioned) |  |  |  |  |  |  |  |
| Team sports | 0.299*** (0.097) | 0.171 (0.107) | 0.168 (0.113) | 0.332*** (0.093) | 0.179* (0.104) |  |  |
| Volunteering | 0.707*** (0.103) | 0.345*** (0.106) | 0.474*** (0.112) | 0.884*** (0.096) | 0.328*** (0.108) |  |  |
| Achievement in tert. edu. (ref. = none mentioned) |  |  |  |  |  |  |  |
| Graduated cum laude | 0.132 (0.154) | 0.302* (0.155) | 0.249 (0.154) | 0.040 (0.151) | 0.205 (0.162) |  |  |
| International experience | 0.261* (0.158) | 0.292* (0.154) | 0.431*** (0.161) | 0.341** (0.143) | 0.346** (0.146) |  |  |
| Mother tongue perceived as excellent | 0.072 (0.084) | 0.033 (0.094) | 0.118 (0.098) | −0.035 (0.085) | 0.029 (0.091) |  |  |
| B. JOB REQUIREMENTS |  |  |  |  |  |  |  |
| Req. level of education: high | −0.200 (0.185) | −0.165 (0.179) | −0.229 (0.183) | −0.204 (0.182) | −0.147 (0.175) |  |  |
| Req. written communication: high | −0.105 (0.147) | −0.064 (0.139) | −0.219 (0.147) | −0.125 (0.146) | −0.146 (0.132) |  |  |
| Req. type of labour: service | 0.225 (0.139) | −0.053 (0.136) | −0.165 (0.145) | −0.018 (0.141) | −0.195 (0.135) |  |  |
| C. PARTICIPANT CHARACTERISTICS |  |  |  |  |  |  |  |
| Female | 0.078 (0.145) | 0.033 (0.140) | −0.113 (0.153) | −0.007 (0.147) | 0.033 (0.135) |  |  |
| Age (c) | −0.011 (0.007) | −0.009 (0.006) | −0.005 (0.007) | 0.061 (0.007) | −0.009 (0.006) |  |  |
| Language sensitivity (c) | 0.020 (0.038) | −0.004 (0.038) | −0.064 (0.042) | −0.010 (0.040) | −0.013 (0.038) |  |  |
| D. MEDIATING PERCEPTIONS |  |  |  |  |  |  |  |
| Perceived as hard-working |  |  |  |  |  | 0.041 (0.070) |  |
| Perceived as organised |  |  |  |  |  | 0.100 (0.071) |  |
| Perceived as thorough |  |  |  |  |  | 0.138* (0.073) |  |
| Perceived as being responsible |  |  |  |  |  | 0.026 (0.063) |  |
| Perceived as systematic |  |  |  |  |  | −0.092 (0.072) |  |
| N | 1,335 |  |  |  |  |  |  |
| Notes. Abbreviations used: s (scale consisting of multiple items), ref. (reference category), req. (required), and tert. edu. (tertiary education). The presented statistics are coefficient estimates and their standard errors in parentheses for the mediation model outlined in Subsection 3.2. Standard errors are corrected for clustering of the observations at the participant level. ***, ** and * indicate significance at the 1%, 5%, and 10% levels, respectively. | | | | | | | |

| Appendix Table 5 continued (2): Extended mediation analysis with interview probability as the outcome and perception items as mediators | | | | | |
| --- | --- | --- | --- | --- | --- |
|  | **Mediators** |  |  |  | **Interview probability** |
|  | **Perceived**  **problem-solving ability** | **Perceived**  **capacity to learn quickly** | **Perceived**  **intelligence** | **Perceived**  **knowledgeability** |  |
| 1. APPLICANT CHARACTERISTICS |  |  |  |  |  |
| Spelling errors (ref. = none) |  |  |  |  |  |
| Two errors | −0.210** (0.091) | −0.389*** (0.092) | −0.571*** (0.092) | −0.427*** (0.091) | −0.247** (0.109) |
| Five errors | −0.704*** (0.104) | −0.881*** (0.109) | −1.323*** (0.117) | −1.094*** (0.113) | −0.664*** (0.123) |
| Female | 0.372*** (0.077) | 0.262*** (0.081) | 0.261*** (0.083) | 0.423*** (0.088) | −0.094 (0.098) |
| Age of graduation (ref. = foreseen age) |  |  |  |  |  |
| One year later | 0.179* (0.108) | 0.204* (0.107) | 0.068 (0.111) | 0.192* (0.104) | −0.010 (0.107) |
| Two years later | 0.192** (0.092) | −0.022 (0.097) | −0.101 (0.099) | 0.134 (0.100) | 0.030 (0.102) |
| Student work (ref. = none mentioned) |  |  |  |  |  |
| On the weekends | 0.255*** (0.093) | 0.263*** (0.090) | 0.195** (0.097) | 0.102 (0.091) | 0.016 (0.118) |
| During holidays | 0.175* (0.092) | 0.114 (0.092) | 0.140 (0.100) | 0.137 (0.099) | 0.013 (0.100) |
| Hobbies (ref. = none mentioned) |  |  |  |  |  |
| Team sports | 0.243*** (0.091) | 0.371*** (0.89) | 0.196** (0.098) | 0.291*** (0.098) | −0.020 (0.109) |
| Volunteering | 0.471*** (0.086) | 0.570*** (0.99) | 0.431*** (0.097) | 0.462*** (0.099) | 0.167 (0.115) |
| Achievement in tert. edu. (ref. = none mentioned) |  |  |  |  |  |
| Graduated cum laude | 0.140 (0.144) | 0.335** (0.155) | 0.726*** (0.154) | 0.366** (0.144) | −0.201 (0.167) |
| International experience | 0.289** (0.138) | 0.534*** (0.155) | 0.887*** (0.153) | 0.651*** (0.140) | −0.125 (0.164) |
| Mother tongue perceived as excellent | 0.066 (0.079) | 0.196** (0.084) | 0.142 (0.092) | 0.122 (0.089) | 0.036 (0.092) |
| B. JOB REQUIREMENTS |  |  |  |  |  |
| Req. level of education: high | 0.057 (0.172) | 0.098 (0.185) | 0.175 (0.186) | 0.132 (0.180) | 0.079 (0.213) |
| Req. written communication: high | −0.058 (0.133) | −0.100 (0.138) | −0.237 (0.145) | −0.352** (0.148) | −0.453** (0.179) |
| Req. type of labour: service | −0.153 (0.130) | −0.164 (0.132) | −0.225 (0.139) | −0.181 (0.140) | 0.095 (0.175) |
| C. PARTICIPANT CHARACTERISTICS |  |  |  |  |  |
| Female | 0.131 (0.132) | 0.048 (0.136) | 0.038 (0.144) | 0.141 (0.145) | 0.017 (0.176) |
| Age (c) | −0.007 (0.006) | −0.008 (0.006) | −0.004 (0.007) | −0.000 (0.006) | 0.001 (0.008) |
| Language sensitivity (c) | −0.021 (0.040) | −0.035 (0.043) | −0.076* (0.042) | −0.097** (0.039) | −0.050 (0.055) |
| D. MEDIATING PERCEPTIONS |  |  |  |  |  |
| Perceived problem-solving ability |  |  |  |  | −0.019 (0.071) |
| Perceived capacity to learn quickly |  |  |  |  | 0.058 (0.074) |
| Perceived intelligence |  |  |  |  | 0.103 (0.098) |
| Perceived knowledgeability |  |  |  |  | 0.343*** (0.083) |
| N | 1,335 |  |  |  |  |
| Notes. Abbreviations used: s (scale consisting of multiple items), ref. (reference category), req. (required), and tert. edu. (tertiary education). The presented statistics are coefficient estimates and their standard errors in parentheses for the mediation model outlined in Subsection 3.2. Standard errors are corrected for clustering of the observations at the participant level. ***, ** and * indicate significance at the 1%, 5%, and 10% levels, respectively. | | | | | |

| Appendix Table 6. Extended mediation analysis: percentages of spelling error’s effects on hiring outcomes explained by individual items | | | | | | | | |
| --- | --- | --- | --- | --- | --- | --- | --- | --- |
|  | **Two spelling errors** | | | | **Five spelling errors** | | | |
| **Mediators** | **Percentage of spelling error’s effect on interview probability explained by mediators [p-value]** | | **Percentage of spelling error effect on hiring probability explained by mediators [p-value]** | | **Percentage of spelling error’s effect on interview probability explained by mediators [p-value]** | | **Percentage of spelling error’s effect on hiring probability explained by mediators** [**p-value]** | |
| A. PERCEIVED INTERPERSONAL SKILLS | | | | | | | | |
| Perceived quality of communication | 4.2% [0.351] | | 3.7% [0.446] | | 4.9% [0.252] | | 4.7% [0.272] | |
| Perceived quality of communication during a job interview | **11.4%** [0.004] | | **6.9%** [0.049] | | **12.8%** [0.000] | | **8.8%** [0.017] | |
| Perceived ability to get along with others encountered on the job | −0.1% [0.598] | | −0.4% [0.787] | | **−2.2%** [0.005] | | −1.8% [0.386] | |
| Perceived pleasure of interacting with the applicant | 3.0% [0.503] | | 3.8% [0.329] | | 4.1% [0.278] | | 5.9% [0.080] | |
| 1. PERCEIVED CONSCIENTIOUSNESS |  |  |  |  |  |  |  |  |
| Perceived as working hard | 1.2% [0.425] | | 0.1% [0.846] | | 1.3% [0.362] | | 0.3% [0.839] | |
| Perceived as working in an organised manner | **5.8%** [0.000] | | 4.7% [0.134] | | **5.8%** [0.000] | | **5.5%** [0.045] | |
| Perceived as working thoroughly | **14.4%** [0.000] | | **19.5%** [0.013] | | **12.1%** [0.000] | | **18.3%** [0.000] | |
| Perceived as working systematically | 1.1% [0.685] | | 1.0% [0.731] | | 1.4% [0.731] | | 1.4% [0.756] | |
| Perceived as being responsible | **−5.2%** [0.032] | | 0.1% [0.983] | | **−5.7%** [0.001] | | 0.1% [0.983] | |
| 1. PERCEIVED MENTAL ABILITIES |  |  |  |  |  |  |  |  |
| Perceived problem−solving ability | −0.0% [0.757] | | 1.6% [0.543] | | −7.2% [0.794] | | 2.5% [0.356] | |
| Perceived capacity to quickly learn new skills | 3.2% [0.334] | | 2.4% [0.213] | | 2.8% [0.306] | | 2.4% [0.174] | |
| Perceived intelligence | **8.1%** [0.027] | | 3.2% [0.427] | | **7.4%** [0.044] | | 3.4% [0.416] | |
| Perceived knowledgeability | **20.1%** [0.021] | | **17.6%** [0.013] | | **20.3%** [0.001] | | **20.1%** [0.001] | |
| N | 1,335 | | | | | | | |
| Notes. Abbreviation used s (scale consisting of multiple items). P-values are corrected for clustering of observations at the participant level. Percentages related to p-values below 5% are in bold. | | | | | | | | |
