# Supplementary material for: Costly mistakes: Why and when spelling errors in resumes jeopardise interview chances
Source: PLoS One. 2023 Apr 5;18(4):e0283280. doi: 10.1371/journal.pone.0283280 (PMC10075394; doi:10.1371/journal.pone.0283280)
Supplement: Attachment — Submitted filename: REVISION PONE-D-22-19619 Response to referees.docx [file pone.0283280.s002.docx]

Revision of “Costly Mistakes: Why and When Spelling Errors in Resumes Jeopardise Interview Chances” for PLOS ONE **│ Response to Reviewers**

We would like to thank the reviewers for their constructive comments. We have responded to each concern of the reviewer. In this document, we give a systematic account of our responses. The original comments are shaded in grey.

**Reviewer 1**

Comment reviewer. “This paper assesses the effect of spelling errors on the interview chances of applicants. The authors conducted a vignette online experiment with real recruiters as participants. To obtain causal estimates they randomly allocated spelling errors to job applications. Results show an interview penalty that is stronger for blue-collar jobs and in case of a higher number of mistakes. The paper is well-written and competently executed. Here are my main comments.”

Response. We thank the reviewer for their appraisal of our work!

Comment reviewer. “The authors rely on a vignette experiment to assess the hiring behavior of recruiters. This opens the question of external validity i.e. do we expect the recruiters to behave similarly in a controlled setting observed by the researchers compared to a real job hiring setting? I would appreciate a discussion on this and also what the previous literature said. An alternative more real-world experiment that they could have done is to send fictitious CVs to real job vacancies. Therefore, it is relevant to compare the pro and cons of the vignette study over the CV experiment and whether these two methods tend to produce compatible results.”

Response. We thank the reviewer for this comment. By further discussing our methodology of choice (and alternatives), the paper is more embedded in the broader literature on hiring experiments. Although we thoroughly discussed the specific measures we took to maximise our study’s external validity, we now frame the matter based on the conclusions from a recent methodological review from Treischl and Wolbring (2022). We have adapted the manuscript accordingly.

From page 8, line 146 onwards:

*“The main limitation of survey experimental designs is their hypothetical nature. More concretely, they measure behavioural intentions rather than actual behaviour. Nevertheless, intentions are a core proxy of behaviour (Ajzen,1991). Regarding the predictive validity of survey experiments, Hainmueller, Hangarter and Yamato (2015) illustrate that survey experiments can predict real-world behaviours. Indeed, in their review Treischl and Wolbring (2022) discuss the validity of survey experiments and suggest that it tends to be the highest when engaged respondents familiar with the decision process (here: hiring) complete tasks that remain faithful to the real-world context, for instance, by providing realistic vignette content.*

*A common alternative to study hiring processes are correspondence experiments where fictitious applications are sent to actual vacancies (see Lippens, Vermeiren and Baert (2023) for an overview). However, whereas correspondence study actual hiring behaviours, they are limited in the sense that they do not allow researchers to survey the decision-maker – which is a crucial contribution of our current study. Applications of survey and correspondence experiments in the study of hiring discrimination are therefore complementary in nature. For instance, both vignette (Van Borm, Burn & Baert, 2021) and correspondence (Lippens et al., 2023) experiments report evidence for ageism. However, whereas correspondence experiments provide estimates in a field setting, vignettes explain ageism through its driving perceptions.”*

Comment reviewer. “After cleaning the dataset, the authors remain with 445 professionals in their samples. What is the response rate to the online survey and the selection/cleaning rate? Can this affect the external validity of the finding?”

Response. Thank you for your question. We can certainly provide some additional information on the data collection procedure. The current study was launched as part of a broader research project involving multiple data collections among HR professionals. For the entire body of surveys, a total of 38,432 invitations was sent out. A total of 4,084 professionals (response rate: 10.6%) started one of this wave’s questionnaires – including ours. Respondents were assigned a survey based on their professional experiences in HR. For instance, only individuals reporting hiring experience qualified for our study. More concretely, a total of 1,026 respondents started specifically our survey. After data cleaning (e.g. deleting incomplete responses), we withheld 445 participants (selection rate: 43.4%). To clarify the matter, we have implemented this information in subsection 2.2 of the revised manuscript.

Reflecting on the impact of our data collection on the external validity of our findings, we do not perceive substantial risks. Although the global response rate for the survey is modest (10.6%), the selection rate is substantial (43.4%). We believe these numbers are not uncommon for contemporary survey research in Belgium, especially when reaching out to a broad audience in a large-scale survey wave. In addition, nonresponse only poses a threat to the validity of findings when non-responders differ systematically from responders on variables that affect the study’s outcome (Fowler, 2014). Given that we were able to collect data from a substantial number (400+) of genuine HR-professionals – instead of small student samples, as is commonly the case in experimental studies – we believe that our data provides us with valuable insights into the population of (Flemish) HR-professionals.

Fowler, F.J., Jr.: Survey research methods. Sage Publications, Los Angeles (2014)

From page 13, line 263 onwards:

*“In April 2020, our vignette experiment was integrated into a large-scale online survey wave for different research projects. In total, 38,432 invitations were sent to genuine HR-professionals living in Flanders. Panel members received an invitation and two weeks later they were sent a single reminder. Hence, a total of 4,084 professionals started one of the wave’s surveys (global response rate: 10.6%). However, only the participants indicating hiring experience qualified for our study. Consequently, 1,026 surveys were started for the current study. Next, participants were withheld in our final study sample when they (i) responded affirmatively to a question on recent hiring experience (‘In the past year, were you responsible for recruiting candidates (applicants)’), (ii) completely filled out the survey and (iii) passed a manipulation check (i.e., responding affirmatively to the question ‘Did the applicants you judge differ in language care?’ near the end of the experiment). After applying these criteria, we withheld 445 professionals and 1,335 vignette observations (selection rate: (445/1,026)*100 = 43.4%).”*

Comment reviewer. “The authors have other control variables that could be used to enhance the efficiency of the estimates (e.g., participant mother tongue, nationality, education level, and hiring tenure). It seems to me that only for a subset of covariates was used in the final regression (age, gender, language sensitivity), I would therefore like to see the results if all the control variables are included.”

Response. Following the reviewer’s request, we have rerun the analyses including all control variables. The results displayed in the Table 4 bis below demonstrate that including additional control variables does not change our results significantly. We did not include all control variables in our original analyses for three reasons. First, we wish to avoid making the models unnecessarily complex. Second, few variables did not have sufficient observations to conduct meaningful heterogeneity analyses (e.g. Flemish recruiters with a non-Belgian nationality). Third, some of the recruiter-side variables were already utilised to conduct robustness checks (e.g. the recruiters’ hiring tenure).

| Table 4 bis. Regression results with interview probability as the outcome variable, two-way interactions included for five spelling errors | | |
| --- | --- | --- |
|  | Interview probability | |
|  | (1) | (2) |
| A. APPLICANT CHARACTERISTICS | | |
| Spelling errors (ref. = none) |  |  |
| Two errors | −0.730*** (0.135) | −0.724*** (0.135) |
| Five errors | −1.843*** (0.155) | 0.531 (1.088) |
| Female | 0.365*** (0.121) | 0.356*** (0.121) |
| Age of graduation (ref. = foreseen age) |  |  |
| One year later | 0.172 (0.145) | 0.168 (0.144) |
| Two years later | 0.143 (0.143) | 0.158 (0.140) |
| Student work (ref. = none mentioned) |  |  |
| On the weekends | 0.307** (0.145) | 0.302** (0.147) |
| During holidays | 0.223* (0.133) | 0.214 (0.135) |
| Hobbies (ref. = none mentioned) |  | |
| Team sports | 0.311** (0.142) | 0.297** (0.144) |
| Volunteering | 0.707*** (0.140) | 0.700*** (0.139) |
| Achievement in tert. edu. (ref. = none mentioned) |  |  |
| Graduated cum laude | 0.108 (0.214) | 0.123 (0.217) |
| International experience | 0.434** (0.213) | 0.457** (0.216) |
| Mother tongue perceived as excellent | 0.151 (0.129) | 0.128 (0.129) |
| B. JOB CHARACTERISTICS | | |
| Req. level of education: high | 0.064 (0.273) | 0.060 (0.276) |
| Req. written communication: high | −0.657*** (0.226) | −0.654*** (0.227) |
| Req. type of labour: service | 0.045 (0.219) | 0.008 (0.220) |
| C. PARTICIPANT CHARACTERISTICS |  | |
| Female | 0.133 (0.228) | 0.094 (0.234) |
| Age (c) | 0.002 (0.011) | −0.003 (0.011) |
| Language sensitivity (c) | −0.114* (0.067) | −0.018 (0.071) |
| Nationality: non-Belgian | −0.588 (0.486) | −0.640 (0.552) |
| Mother tongue: non-Dutch | −0.488 (0.531) | −0.740 (0.745) |
| Education level (ref. secondary education) |  |  |
| Tertiary education bachelor | 0.063 (0.438) | 0.181 (0.451) |
| Tertiary education master | −0.003 (0.438) | 0.092 (0.446) |
| Hiring tenure (ref. less than 1 year) |  |  |
| 1 to 5 years | 0.415 (0.443) | 0.616 (0.474) |
| More than 5 years | 0.242 (0.439) | 0.415 (0.468) |
| Five errors × Female |  | 0.102 (0.266) |
| Five errors × Age |  | 0.013 (0.014) |
| Five errors × Language sensitivity |  | −0.289*** (0.084) |
| Five errors × Nationality: non-Belgian |  | 0.105 (0.579) |
| Five errors × Mother tongue: non-Dutch |  | 0.752 (0.985) |
| Five errors × Tertiary education bachelor |  | −0.355 (0.450) |
| Five errors × Tertiary education master |  | −0.284 (0.434) |
| Five errors × 1 to 5 years of tenure |  | −0.554 (0.582) |
| Five errors × More than 5 years of tenure |  | −0.493 (0.559) |
| N | 1,335 | |
| Notes. Abbreviations used: c (continuous variable), ref. (reference category), req. (required), and tert. edu. (tertiary education). The presented statistics are coefficient estimates and their standard errors in parentheses. Standard errors are corrected for clustering of the observations at the participant level. ***, ** and * indicate significance at the 1%, 5%, and 10% levels, respectively. | | |

Comment reviewer. “It should be specified what "α" is (e.g. line 344) as it is not mentioned until that point”

Response. We used the alpha symbol to represent the Cronbach’s alpha reliability of the language sensitivity scale. We have now clarified this in the manuscript by explicitly naming it “Cronbach’s α” to avoid any confusion.

Comment reviewer. “I found it interesting that women tend to be hired more than men (see e.g. Table 4). Since also gender was randomized in the application, this may also be interpreted as causal. Do the authors have an explanation for that, for example the type of jobs chosen (this brings back the question of external validity)?”

Response. We agree with the reviewer that, given the experimental design, a causal interpretation can be given to the relationship between candidate gender and the hiring probabilities. It is not uncommon to identify small gender effects in hiring experiments. After all, the high number of observations per participant allows researchers to detect subtle differences in vignette manipulations. Example studies are the work from Van Belle and colleagues (2018) where women were assigned slightly higher interview probabilities (context: counter assistant) and the study from Sterkens and colleagues (2021) where women were at a slight disadvantage (context: multiple jobs).

One likely explanation is that the job contexts of hiring assignments elicits recruiters’ subtle gender preferences – these effects are generally (very) small. We believe that, for the current study, this does not pose a threat to the validity of our results because we possessed strong indications (O*NET database) that our current, relatively broad, job selection correctly reflected the job characteristics of interest (e.g. required written communication skills).

Comment reviewer. “The main outcome is "the probability they would invite an applicant for a first job interview (hereafter referred to as ‘interview probability’)" (line 290). I have therefore not cleared why the coefficient of B=-1.85 is translated to -18.5 percentage points.”

Response. The interview probability measure is rated on a scale ranging from 0 to 10. This measure’s average across participants therefore also ranges from 0 to 10 (as a continuous scale). By dividing the scales’ values by 10, we calculate at a classical probability (between 0 and 1) and after multiplying this by 100 we have a probability in percentage terms. The difference between two percentages (i.e. the probability of being invited for two separate groups) then provides a value that can be interpreted as a difference in percentage points.

Comment reviewer. “The authors compare their results on the total penalty to Toorenburg et al. (2015), which uses white collar and five errors only. The authors should compare Toorenburg to their own estimates on white collar and five errors only.”

Response. We thank the reviewer for their insightful comment! The suggested comparison would be the most sensible given the similar elements in both studies. Consequently, we have conducted a separate analysis and reported the result. More specifically, we find a 12.2 percent point decrease in interview probabilities for 5 spelling errors in white collar occupations. In comparison, Van Toorenburg and colleagues (2015) report a 13.7 percent point decrease in hireability ratings for five errors in a similar occupation.

From page 19, line 416 onwards:

*“When we compare our study’s subsample estimate Our for estimated effect of five spelling errors in the context white-collar occupations (–12.2 percent points), we find that it is well comparable to a study among HR recruiters by van Toorenburg et al. (2015) reporting a 13.7 percent points decrease in hireability ratings of HR specialists for resumes featuring five spelling errors.”*

Comment reviewer. “I would clarify the sentence at line 401, by modifying it as follows (or something similar): "As participants could vary in unobserved, confounding variables, a causal interpretation of the interactions regarding the PARTICIPANT CHARACTERISTICS is inappropriate."”

Response. Thank you. We followed the reviewer’s suggestion and it improved the overall clarity of the paragraph.

Comment reviewer. “The authors mention at the beginning of the paper that one of their main contributions is to estimate the effect also for blue-collar occupations since the previous literature mostly focused on white-collar jobs. I would stress throughout the paper that when they mentioned “jobs with high educational requirement" they actually mean white-collar jobs.”

Response. Following the reviewer’s request, we have further clarified the matter by repeating our choice for white collar jobs throughout the manuscript.

Comment reviewer. “missing space on page 13, line 275”

Response. Thank you. While revising our manuscript we have sought to remove any remaining stylistic errors from the manuscript.

**Reviewer 2**

Comment reviewer. “This is a well-written research article dealing with an interesting topic. Both the experimental design and empirical analysis are reasonable, and the results are clear.”

Response. We would like to thank the reviewer for their kind words.

Comment reviewer. “The authors introduce spelling errors in the fictitious resumes. However, language errors come in many forms and could be both pure spelling errors and more structural grammatical errors. It would be nice if the authors could elaborate more on their choice of spelling errors. How common are the spelling errors used? Will standard word-processing software detect these spelling errors, and hence make them less likely to occur in real-world resumes? Would an alternative design with grammatical error be more interesting? The authors should discuss these issues briefly in the main text, and include the actual spelling-errors used in an online appendix. The authors should also include an example of the vignette in the online appendix.”

Response. In the developmental phase of our experimental resume materials, we were limited in the number and type of errors that were realistic to implement because (1) resumes seldom contain long, complex sentences and (2) experimental set-ups require that resume templates (and their content) are comparable except for the manipulated dimensions. Therefore, in the current study we mainly focussed on ‘pure’ spelling errors through incorrect verb conjugations or improper applications of phonetic spelling. Hence, our design did not feature complex grammatical errors. As we explain in subsection 2.1, we included common spelling errors. For instance, we implemented violations of the Dutch ‘dt-rule’ (i.e. an incorrect verb conjugation), which is the most common error in Dutch according to Heyman and colleagues (2018). Moreover, approximately 20 pretesters (all native Dutch-speaking) recognised the errors in the resumes and evaluated them as realistic.

Word-processing software should be able to identify many instances of spelling errors. Nevertheless, in line with the impressions from our pretesters (as a source of anecdotical evidence), these errors still occur in real-life hiring contexts. As an example: despite the fact that violations of the dt-rule (one of the error types we implemented) can often be spotted by software – from our own experience – it remains the most common spelling error in Dutch (Heyman et al., 2018). Although software could prevent many of the errors from occurring, the possibility remains that individuals do not use the software (for instance, when using external resume formatting tools online). Alternatively, the word-processing software could be used improperly. An example of improper use could be when texts contain combinations of Dutch and English (professional terminology) and the user does not adjust the reviewing language accordingly.

It would be valuable to conduct a similar study with (more complex) grammatical errors. If we were to do so as researchers, we would recommend to develop experimental materials in the shape of cover letters accompanying resumes. In a letter format it could be more feasible to include several and more complex grammatical errors. However, when manipulating different cover letters and presenting them within-subject, it would be challenging to maintain a balance between exercising experimental control (‘except for the manipulations, the materials should be comparable’) and avoiding unrealistic and repetitive letter content.

We agree with the reviewer that sharing some extracts of the experimental materials could improve the readers’ understanding of our work. Therefore, we have added both a record of the errors and an example vignette to the appendix.

From page 9, line 185 onwards:

*“Errors in resumes violated the rules of spelling by, for example, incorrectly conjugating verbs or incorrectly spelling words phonetically. Incorrect conjugations of verbs resulting from an erroneous implementation of the ‘dt-rule’ are the most common spelling errors in Dutch (Heyman, Vuli, Laevaert & Moens, 2018). Other examples of implemented errors are: ‘manlijk’ (correct: ‘mannelijk’; male (English)) and ‘vollybal’ (correct: volleybal; volleyball (English)). Cross-validation of errors was performed by two native Dutch-speaking researchers and twenty pretesters (i.e., pretesters (i) recognised the errors in resumes and (ii) evaluated the proposed errors as realistically occurring). The complete record with Dutch spelling errors used in the experiment is displayed in Appendix Figure 1. Theoretically, word-processing software should be able to trace many errors we implemented in our experimental materials – and these errors could be avoided in practice. However, in line with our pretesters’ evaluation of the errors as ‘realistically occurring’, applicants might not use word-processing software at all (e.g. when formatting a resume in online tools) or incorrectly (e.g. failing to adjust the settings when languages are combined in documents (e.g. Dutch and English)).”*

From page 30, line 688 onwards:

*“Another avenue for future research could be to investigate different types of language errors. Indeed, whereas our design featured common spelling errors such as incorrect verb conjugations and improper phonetic spelling, we did not investigate complex grammatical errors. Experimental manipulations of cover letters that accompany (fictious) resumes could provide an ecologically valid context to study the impact of grammatical errors on job market prospects.”*

Comment reviewer. “The presentation of the results could be improved. Table 4 contains the main results, but this table is rather large and somewhat difficult to read. I would suggest that the authors include more guidance to the reader in the main text about where the reader should look in the table (i.e., clearer references to the panels and columns when they discuss different estimates/results). Also, the results for the interactions with two spelling errors should be included in an online appendix.”

Response. We can understand the difficulties the reviewer experienced when consulting Table 4 because, indeed, the number of parameters in the different moderation models resulted in rather large tables. Following the reviewer’s suggestion, we have included additional guidance for the reader by mentioning the table, column and panel for each estimate we discuss from these analyses. Next, as requested by the reviewer, the results of the analyses with two spelling errors have been added to the manuscript’s appendix and the text has been corrected (i.e. for a manual error) accordingly.

Comment reviewer. “A major limitation with the study is the hypothetical nature of the methodology used; i.e. the participants know that they answer a survey and that their answers have no real-world consequences. This is mentioned and discussed in the concluding section, but this limitation is so important that it should be highlighted earlier (i.e., briefly in the introduction and in the section on method).”

Response. We acknowledge that the study’s main limitation is the hypothetical nature of vignette experiments. Following the reviewer’s request, we have discussed this matter more thoroughly in both the method section and the introduction.

From page 7, line 125 onwards:

*“Whereas the rigour of vignette experiment is at the core of this study’s strengths, the hypothetical nature of participants’ decisions is our methodology’s main limitation.”*

From page 8, line 146 onwards:

*“The main limitation of survey experimental designs is their hypothetical nature. More concretely, they measure behavioural intentions rather than actual behaviour. Nevertheless, intentions are a core proxy of behaviour (Ajzen,1991). Regarding the predictive validity of survey experiments, Hainmueller, Hangarter and Yamato (2015) illustrate that survey experiments can predict real-world behaviours. Indeed, in their review Treischl and Wolbring (2022) discuss the validity of survey experiments and suggest that it tends to be the highest when engaged respondents familiar with the decision process (here: hiring) complete tasks that remain faithful to the real-world context, for instance, by providing realistic vignette content.”*

*A common alternative to study hiring processes are correspondence experiments where fictitious applications are sent to actual vacancies (see Lippens, Vermeiren and Baert (2023) for an overview). However, whereas correspondence study actual hiring behaviours, they are limited in the sense that they do not allow researchers to survey the decision-maker – which is a crucial contribution of our current study. Applications of survey and correspondence experiments in the study of hiring discrimination are therefore complementary in nature. For instance, both vignette (Van Borm, Burn & Baert, 2021) and correspondence (Lippens et al., 2023) experiments report evidence for ageism. However, whereas correspondence experiments provide estimates in a field setting, vignettes explain ageism through its driving perceptions.”*

**Reviewer 3**

Comment reviewer. “I found this paper to be a very compelling and clear read. The authors tackle an interesting question in a very competent and thorough manner. I found some grammar mistakes : ) but they were too few to remember. I would encourage them to pursue a field experiment as a complement to the lab experiment described in their work as it would provide external validation to their results. But that would be the goal of another paper.

I have one small suggestion: to describe how long it took the recruiters to evaluate their three applications and complete the follow-up survey.”

Response. We thank the reviewer for their evaluation of our work and the suggestion for future research in this field. In response to one of the other reviewer’s comment we briefly discuss the option of conducting a field experiment (and the matter of external validity) in the revised manuscript.

In response to your suggestion: we fear that we were unable to retrieve this information from our dataset export. As we explain in subsection 2.2, this survey was part of a large-scale online survey wave for different research projects. Therefore, we can no longer separate the time it took participants to complete our study from the duration of the broader survey. However, based on our experience with similar vignette designs, we informed participants in the introduction of our study it would take them approximately 15 minutes.

From page 30, line 688 onwards:

*“Another avenue for future research could be to investigate different types of language errors. Indeed, whereas our design featured common spelling errors such as incorrect verb conjugations and improper phonetic spelling, we did not investigate complex grammatical errors. Experimental manipulations of cover letters that accompany (fictious) resumes could provide an ecologically valid context to study the impact of grammatical errors on job market prospects.”*

From page 8, line 155 onwards:

*“A common alternative to study hiring processes are correspondence experiments where fictitious applications are sent to actual vacancies (see Lippens, Vermeiren and Baert (2023) for an overview). However, whereas correspondence study actual hiring behaviours, they are limited in the sense that they do not allow researchers to survey the decision-maker – which is a crucial contribution of our current study. Applications of survey and correspondence experiments in the study of hiring discrimination are therefore complementary in nature. For instance, both vignette (Van Borm, Burn & Baert, 2021) and correspondence (Lippens et al., 2023) experiments report evidence for ageism. However, whereas correspondence experiments provide estimates in a field setting, vignettes explain ageism through its driving perceptions.”*
